# Internet Addiction: The Role of Parental Care and Mental Health in Adolescence

**DOI:** 10.3390/ijerph182412876

**Published:** 2021-12-07

**Authors:** Carmen Trumello, Laura Vismara, Cristina Sechi, Piera Ricciardi, Valentina Marino, Alessandra Babore

**Affiliations:** 1 Department of Psychological, Health and Territorial Sciences, University “G. d’Annunzio” of Chieti, Via dei Vestini, 66100 Chieti, Italy; c.trumello@unich.it (C.T.); piera_ricciardi@yahoo.it (P.R.); valentmarino@gmail.com (V.M.); 2 Department of Pedagogy, Psychology, Philosophy, University of Cagliari, 09124 Cagliari, Italy; vismara@unica.it (L.V.); cristina.sechi@unica.it (C.S.)

**Keywords:** mental health, adolescents, parents, parental care, Internet addiction, structural equation model

## Abstract

Internet addiction in adolescence is a social issue which is being increasingly discussed worldwide. Hence, deepening the knowledge of its development is necessary to prevent short- and long-term negative outcomes. This study involving 266 adolescents (mean age = 16.1 years, SD = 1.4) aimed at analyzing the relationship between quality of parental care, Internet addiction and adolescents’ mental health, by means of the following self-report tools: the Parental Bonding Instrument, the Internet Addiction Test, and the Strengths and Difficulties Questionnaire. The findings, collected through structural equation model analyses, showed that perceived care from both father and mother had significant indirect effects on Internet addiction problems through adolescents’ mental health problems. Furthermore, Internet addiction problems were demonstrated to be negatively associated with maternal care but not with paternal care. The study provides empirical support to the need of family-based prevention and intervention programs to take care of Internet addiction.

## 1. Introduction

The Internet plays an important role, as it gives youths the possibility to have experience of and explore critical issues, such as autonomy, identity, and sexuality [1,2]. In the last decade, there has been a huge expansion of new kinds of information and communication technology, as social media, smartphones, personal computers, and other devices [3]. Adolescents and young adults are the main users of these instruments [4,5], with the primary purpose of social interaction and interpersonal communication [6]. Although these new technologies are used by teenagers to address their developmental duties, existing research has shown that they could hinder their growth. The Internet may be used excessively by adolescents or in a maladaptive manner, in particular to manage psychological hardship [7] and negative emotions linked with difficult parental and peer relationships [8]. An excessive and uncontrolled use of the Internet may present many negative influences and potential risks [9,10]. Internet addiction (IA) can be defined as the inability to control Internet use despite the negative effects [11]. IA, also referred to as problematic Internet use or pathological Internet use, is accompanied by withdrawal symptoms and adverse consequences [12,13]. Several studies have indicated that IA may produce negative outcomes on adolescents’ well-being, social relationships, and school performance [14,15,16]. This type of addiction involves a decreasing sense of importance of activities not implicating the use of the Internet, high levels of nervousness and aggression in the case of Internet deprivation, with a gradual worsening of personal, social, and familiar life [17]. 

To tackle and prevent problems related to the Internet use in adolescence, it is necessary to understand IA development [18]. Quality of parental care and parent–child relationships [19,20] offer a valuable model for understanding the risky or protective role played by parenting in the development of adolescent’s behavior, and can also provide an important contribution to understanding the pathogenesis of IA [21]. Following Armsden and Greenberg [22], the adolescents’ perceptions of parental relationship reflect their support from and emotional closeness to attachment figures, in terms of warmth, trust in their accessibility and responsiveness, involvement, and nurturance. Adolescents that perceive their parents as emotionally unavailable, not supportive, and cold, may excessively use the Internet to look for alternative social support [4,23]. There is considerable evidence that parent–child relationship quality plays an important role in IA development. In particular, negative parent–child relationships [24,25] and low parental care [26] have been found to be associated with IA. A good relationship with parents, effective parenting and parental emotional availability could provide adolescents with a stable and safe environment for growth and help prevent them from engaging in at-risk behaviors such as Internet abuse. Results of a recent study [27] showed that lower levels of maternal emotional availability predicted higher IA. This association was not confirmed for paternal emotional availability.

An important body of research examined the relation between parental care and adolescents’ mental health problems. Attachment security, a positive quality of parental relationship, and parents’ availability may serve as protective factors for adolescents’ mental health [28]. Growing evidence suggests that poor parental care quality is an important predictor of adolescents’ maladjustment. Previous studies have identified relationships of various parenting practices and adolescents’ internalizing [29] and externalizing problems [30]. The existing literature on this theme reports a significant link between parental care and a wide range of dysfunctional outcomes including depression [31,32], anxiety [33,34], and several addictive disorders [35,36].

Research has reported significant connections between IA and emotional/psychosocial problems [37,38], including depressive/anxiety symptoms [39], and psychosomatic symptoms [40]. Although the relationship between IA and mental health could be considered bi-directional, two recent longitudinal studies showed that the latter was predictive of the former; research on adolescent girls [41] detected that baseline depressive symptoms were predictive of changes in problematic social media use (but baseline problematic social media use did not predict changes in depressive symptoms), and another study [42] found hyperactivity/inattention and self-esteem problems to be important for the development of internet gaming disorder.

Several studies highlight that isolation feelings, anger or detachment within parental relationships may lead adolescents to cope with these negative emotions through excessive Internet use, in order to reduce the distress resulting from unfavorable relational experiences [43]. Ballarotto et al. [44] showed that adolescents’ attachment to parents influences Internet use/abuse and that their psychopathological risk played a moderating role on the relationship between attachment to mothers and Internet use. However, the understanding of risk factors for IA is not complete and few studies have examined the combined, moderating, and modulating effects of parental relationship and adolescents’ adjustment on IA [45].

### The Current Study

The present research aimed to address the lack of studies about the combined associations of the aforementioned factors, investigating the relationship between perceived care from both father and mother, mental health problems and IA among Italian adolescents. Based on the clinical and empirical framework considered above, we hypothesized that both poor perceived maternal and paternal care would be associated with adolescents’ IA problems. We further explored whether adolescents’ mental health problems would mediate the effect of maternal and paternal care on adolescents’ IA problems.

Specifically, we have hypothesized that:

**Hypothesis** **1** **(H1)** . *Maternal care would be negatively associated with adolescents’ IA problems;*

**Hypothesis** **2** **(H2).** *Paternal care would be negatively associated with adolescents’ IA problems;*

**Hypothesis** **3** **(H3).** *Maternal care would be negatively associated with adolescents’ mental health problems;*

**Hypothesis** **4** **(H4).** *Paternal care would be negatively associated with adolescents’ mental health problems;* and

**Hypothesis** **5** **(H5).** *Adolescents’ mental health problems would be positively associated with IA problems.*

Moreover, a further aim was to analyze if there were gender differences in the studied variables. Following previous research [46], we hypothesized higher levels of IA in boys than girls.

## 2. Materials and Methods

### 2.1. Participants

The sample comprised two hundred sixty-six non-referred adolescents. Specifically, the study involved 143 (53.8%) males and 123 (46.2%) females. The age of the participants ranged from 14 to 20 years, with a mean age of 16.1 years (SD = 1.4). Adolescents filled in questions regarding demographic information about their parents and family composition. According to them, fathers’ mean age ranged between 38 and 70 years (M = 49.3 years, SD = 5.5 years), whereas mothers’ mean age ranged between 34 and 70 years (M = 45.9 years, SD = 5.4 years).

Based on a detailed questionnaire developed following ISTAT [47] classification, the median parental income belonged to the Italian middle working class and socio-economic status. All participants lived with both their parents. No incentives were provided to the study participants.

### 2.2. Measures

#### 2.2.1. Demographic Information

Adolescents were asked to give information on their age and gender, as well as on their parents’ age, education, and current employment (separately for fathers and mothers).

#### 2.2.2. Parental Care

Perceived quality of the relationship with fathers and mothers was evaluated by means of the Parental Bonding Instrument (PBI) [48], a commonly used scale assessing adolescents’ perceptions of two spheres of parenting: parental caring and overprotection, separately for father and mother. It consists of 25 statements, 12 of which relate to care (e.g., “appeared to understand my problems and worries”) and 13 to overprotection (e.g., “tried to control everything I did”). Items are rated on a 4-point Likert scale from 0 ‘very unlike’ to 3 ‘very like’. Two scores are obtained, one for care dimension and one for protection dimension, with higher scores suggesting more parental care and overprotection, respectively. In this study, we used the paternal and maternal care subscales of the Italian version of the PBI [49]. The reliability of this scale in the current research was good (Cronbach’s alpha = 0.852).

#### 2.2.3. Adolescents’ Mental Health Problems

The self-report form of the Strengths and Difficulties Questionnaire (SDQ) [50] was used to evaluate social, emotional, and behavioral problems related to mental health in adolescents. It consists of 25 items grouped in five subscales (each composed by five items): conduct problems (CP, e.g., “I usually do as I am told”), emotional symptoms (ES, e.g., “I have many fears, I am easily scared”), peer problems (PP, e.g., “I get along better with adults than with people my own age”), hyperactivity (HY, e.g., “I am restless, I cannot stay still for long”) and prosocial behavior (PB, e.g., “I am helpful if someone is hurt, upset or feeling ill”). Items are rated using the 3-point Likert scale from 0 “Not True” to 2 “Certainly True”. A total difficulty score can be calculated by summing the subscale scores of the negative attributions: CP, ES, PP and HY. The reliability was satisfactory (Cronbach’s alpha = 0.753).

#### 2.2.4. Internet Addiction

The Internet Addiction Test (IAT) [51] is a 20-item self-report measure assessing the extent to which internet use interferes with emotional feelings, sleeping patterns, one’s daily routine, and social life. Dysfunction is assessed on a six-point Likert scale (0 = does not apply to 5 = always), with higher scores denoting a higher level of problems related to internet use. Factor analysis of the Italian IAT by [52] yielded to two factors: “Emotional and cognitive preoccupations with the Internet and social consequences” (ECP) and “Loss of control and interference with daily duties” (LC), both showing good internal consistency and convergent validity. The ECP includes items related to the emotional and cognitive salience of Internet use and obsessive thoughts about Internet when offline, and items concerning the negative social consequences due to Internet use (e.g., “How often do you fear that life without the Internet would be boring, empty, and joyless”). The LC contains items related to unsuccessful attempts to control the amount of time spent online and to the negative consequences of the Internet use on daily functioning (e.g., “How often do you find yourself saying ‘just a few more minutes’ when online?”). In the present research, the reliability was excellent (Cronbach’s alpha = 0.906).

### 2.3. Procedure

The research project was presented in five Italian state high schools, under approval and authorization of the school principals. Students were randomly selected from ten courses of each of the five selected schools. After briefly introducing the goals of the study, students were given consent forms to take home. Parents gave written informed consent for their children and students gave written informed assent for themselves. The procedure and all the instruments used in this study were fully in compliance with the Ethics Code of the Italian Board of Psychology (the regulatory Authority providing the national guidelines for research and clinical practice) and the indications of the Declaration of Helsinki.

No student decided to withdraw from the study. No students’ identifying information was collected. During school hours, research assistants administered the questionnaires to students within the school classrooms. The completion time was approximately 45 min. Data were collected between March and May 2017.

### 2.4. Data Analysis

Multiple imputation (MI) [53] for missing data was applied. First, the data were evaluated to verify that missing values were missing at random (MAR). Then, the degree of missing data was analyzed to guarantee that less than 10% of data were missing across scale scores. The postulation of MAR was met, and the occurrence of missing data across scales (1–3%) was appropriate. Twenty-five multiply imputed data sets were produced. 

Descriptive data for the sample was summarized using means and standard deviations. 

An independent *t*-test was run to verify whether there were gender differences between study variables.

At the multivariate level, the pattern of associations specified by our hypothesized model was analyzed through the two-step procedure suggested by Anderson and Gerbing [54]. Specifically, the measurement model was first examined to evaluate the extent to which each of the latent variables was characterized by its indicators. The measurement model contained two latent variables (mental health problems and Internet addiction) and six observed variables. Specifically, the “mental health problems” latent variable was assessed using the four subscales of SDQ (CP, ES, PP and HY) and the “Internet addiction” latent variable was assessed by two subscales of the Italian IAT (ECP and LC). Following procedures for lower bounds for sample size determined by Westland [55], the minimum sample size for SEM was estimated to be 200 according to an effect size of 0.3, statistical power level of 0.8, two latent variable and six observed variables. Thus, using these criteria, the sample size of the present study (*n* = 266) had sufficient power to test our hypotheses. If the measurement model is accepted, then the constructs of Mental health problems and Internet addiction can be presumed to be well represented by the data. The structural equation model (SEM) was then utilized to assess the hypothesized model that included two latent factors (mental health problems and Internet addiction) and eight observed variables: two supposed observed antecedent variables (paternal and maternal care), one latent mediator variable (mental health problems), and one outcome variable (Internet addiction). 

Before conducting the SEM analyses, study variables were tested for normality [56] using the cut-offs for skewness (absolute value ≥ 2) and kurtosis (absolute value ≥ 7) [57]. The values of skewness (0.43–1.87) and kurtosis (0.68–2.94), revealed a normal distribution of the variable scores. The following indices were used to assess the goodness of fit of the model: chi-square statistics (χ^2^), the Tucker Lewis Index (TLI), the comparative fit index (CFI), the standardized root-mean-square residual (SRMR), and the root-mean square error of approximation (RMSEA), along with its 90% confidence interval (CI). It is recommended that good fit indices for TLI and CFI are greater than 0.90, and for SRMR and RMSEA, less than 0.08 [58,59]. The significance of the effects was analyzed using a 95% bootstrapped confidence interval estimate. Our sample fits about the minimum sample size (*n* = 100) for the model structure with two latent and eight observed variables [55], at an alpha level of 0.03, a power of 0.80, and an anticipated effect size of 0.3.

## 3. Results

### 3.1. Preliminary Analysis

Levene’s test of homogeneity of variances demonstrated that the variance was homogeneous between females and males.

An independent *t*-test revealed that females’ and males’ SDQ scores were significantly different (Table 1). Specifically, female adolescents reported significantly higher scores on the emotional symptoms than male adolescents, while males reported significantly scores on the conduct problem than female adolescents. No significant gender difference was found in the PBI and IAT scales.

### 3.2. Testing the Measurement Model

**Figure 1:**
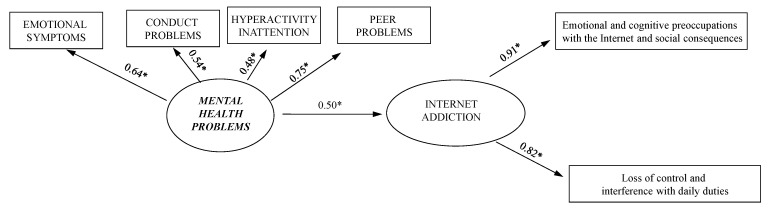
The measurement model and related standardized regression weights, * *p* < 0.001.

The measurement model and related standardized regression weights are reported in Figure 1. Fit for the measurement model was very good (χ^2^ = 13.49, df = 0, *p* = 0.96, CFI = 0.98, TLI = 0.97, RMSEA = 0.05 (90% CI 0.00 to 0.10), SRMR = 0.03). The latent variables were significantly associated with each other (*p* < 0.001). In addition, all the factor loadings were significant (*p* < 0.001), which is evidence for the convergent validity of the indicators. Thus, the measurement model was considered suitable for the following analyses.

### 3.3. Structural Model

As a first step, whether paternal and maternal care and IA problems were associated in absence of mental health problems was investigated.

The direct and negative association between paternal care and IA problems was not significant, whereas the direct and negative link between maternal care and IA problems was significant (−0.07, *p* > 0.05; −0.24, *p* < 0.001, respectively). 

The next step was to test a mediating effect model which estimates the direct effects from paternal and maternal care to IA problems and adds the paths from paternal and maternal care to mental health problems, and from mental health problems to IA problems. 

The result of the mediating effect model was suitable (χ^2^ = 24.55, df = 16, *p* = 0.08, CFI = 0.98, IFI = 0.97, RMSEA = 0.05 (90% [CI]: 0.00 to 0.08), SRMR = 0.03).

**Figure 2:**
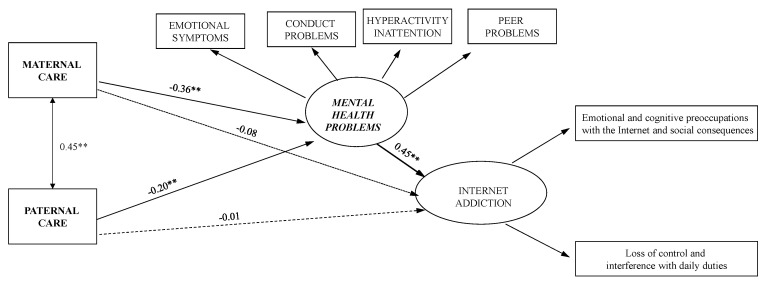
Structural model with standardized path coefficients. ** *p* < 0.01. Note: the dotted line is non-significant paths.

It should be observed that there were no significant direct effects of both paternal and maternal care on IA problems (Table 2; Figure 2). 

Perceived care from both father and mother had significant indirect effects on IA problems through mental health problems (Table 2).

## 4. Discussion

In consideration of the complex interactions among different individual and family variables, the current study aimed at analyzing the relationship between quality of parental care (as perceived by adolescents), Internet addiction (IA) and adolescents’ psychological mental health.

As regards the first hypothesis, our findings showed that poor levels of maternal care were associated with higher IA problems. These results confirm previous scholars [21,44,60,61,62,63]. Indeed, IA may be conceived as a dysfunctional mechanism to face negative and distressful situations [64]. IA may constitute a substitute for the need of emotionally satisfying bonds with parents in the presence of parent–child relationships that are perceived as uncaring and cold [65,66,67].

However, as stated, we found a negative association between maternal care and IA problems, whereas no association emerged with respect to paternal care. It seems that maternal parenting is more influential on the development of IA, consistently with other studies [27,68]. We may speculate that this result is influenced by the fact that mothers are the primary caregivers in Western societies [69], although such a role seems also to be biologically grounded [70].

In line with the parental care perspective [19,20,71], our study also reveals that low maternal and paternal care were related to poor adolescents’ mental health problems, confirming our hypotheses. These findings are in accordance with previous research indicating that the lack of supportive relationships with parents is linked to a higher psychopathological risk in the offspring [72,73].

As regards gender differences, contrary to our expectations, the data showed that gender had no effect on adolescents’ IA problems. These results may suggest that Internet addiction is cross-gender; nevertheless, different temperamental, behavioral, and environmental characteristics may explain the development of problematic internet use in boys and girls that warrant further investigation. Moreover, the study highlighted gender differences in the expression of mental health problems. Confirming previous data [74], the girls of our sample showed higher scores on the emotional problems scale, whereas boys presented higher scores on the conduct problems subscales. Typically, boys have difficulties in inhibiting negative behaviors, controlling impulses, and regulating negative emotions, while girls are more relationally oriented, calm, and able to regulate emotions than boys [75]. Such differences may be due to biological processes [76], as well as environmental factors that may increase vulnerability to psychopathological outcomes [77,78]. For such reasons, it is important to carry on early screening of psychological symptoms. Undeniably, misuse of Internet may worsen psychological symptoms, which, in turn, may trigger Internet addiction itself. In addition, gender should be considered, since differences have been frequently reported in the patterns of IA and its protective and risk factors [79,80,81].

Finally, in addition to the direct effects of maternal care on adolescents’ IA problems, our findings showed that both maternal and paternal care were indirectly related to IA problems through adolescents’ mental health. Therefore, our data highlighted that adolescents who perceive their parents as cold and uncaring are more likely to experience mental health problems which, in turn, are related to more IA problems. These results provide evidence that adolescents’ mental health problems are an important mediator between paternal and maternal care and youth’s IA. Specifically, the onset of IA in both male and female adolescents is mediated by the presence of psychological difficulties in them. However, previous studies highlighted a bi-directional relationship between mental health problems and IA [82,83,84,85], and this possibility cannot be excluded in interpreting our findings. Furthermore, we may not exclude an interaction effect between adolescents’ IA and parents’ Internet use/abuse [86], as this last variable was not investigated in our study. Further longitudinal research should address these issues, as they are particularly relevant to deepen the topic.

Despite the relevance of our results, some methodological limits must be considered in interpreting our results. First, the lack of representativity of the sample; participants were all from the same socioeconomical background and from the same Region of Italy. Most important, as above mentioned, the cross-sectional nature of this study does not allow conclusions about cause-and-effect relations to be drawn; therefore, the associations we found should be confirmed through longitudinal studies. In line with this issue, we cannot interpret the verified associations over time. Moreover, in the evaluation of the complexity of factors that may be entangled in the onset of IA, future studies should also include the evaluation of the relationship with peers, which is particularly salient in this period of the life cycle. Finally, this study was carried out before the beginning of the COVID-19 pandemic and therefore it does not take into consideration the huge changes that the pandemic has produced on parent–child relationships [87,88,89] and Internet addiction [90].

## 5. Conclusions

Our path analysis findings explained some of the potential underlying ways of how the quality of parental care may protect adolescents against their psychological mental health and addictive internet behaviors. Family-based interventions aimed at enhancing parent–child relationships, communication and understanding can be a direction for adolescents’ Internet addiction prevention. Future studies should concentrate on giving evidence on the effectiveness of interventions to reduce Internet addiction.

However, our results also point out the need to assume an integrative perspective, that encompasses both interpersonal and intrapersonal factors. The findings point out the importance of the individual characteristics of the adolescent on the development of at-risk addictive use of the Internet and the complexity of the onset of maladaptive functioning and psychopathological symptoms. Thus, a multifaceted approach that encompasses personal and environmental factors must be embraced to understand the development of adolescent Internet addiction. Furthermore, internet characteristics should also be considered. Future studies focusing on specific sub-types of addiction (e.g., gaming or social media addiction) are warranted to improve the efficacy of preventive and treatment interventions.

Finally, the specific characteristics of the single adolescent, her/his undergoing developmental stage, and family context must be considered to improve the efficacy of interventions.

## Figures and Tables

**Table 1:** Means (SDs) for parental and adolescent ratings.

Scores for Parental Care (PBI)	Total Sample	Males	Females	*t*	*d*
Maternal Care	25.61 (5.20)	25.48 (5.04)	25.76 (5.41)	0.45	0.05
Paternal Care	23.63 (8.15)	24.39 (7.16)	22.74 (9.12)	−1.65	0.20
Scores for adolescents’ mental health problems (SDQ)					
Conduct problems	3.03 (1.57)	3.20 (1.69)	2.83 (1.40)	−1.95 *	0.24
Emotional symptoms	3.89 (2.66)	2.86 (2.23)	5.10 (2.62)	7.53 **	0.92
Peer problems	2.76 (1.74)	2.63 (1.64)	2.91 (1.84)	1.32	0.16
Hyperactivity	4.78 (1.65)	4.73 (1.67)	4.83 (1.62)	0.47	0.06
Scores for adolescents’ Internet addiction problems (IAT)					
Emotional and cognitive preoccupations with the Internet and social consequences	21.63 (7.48)	21.69 (7.72)	21.57 (7.22)	0.45	0.02
Loss of control and interference with daily duties	20.82 (6.56)	21.21 (6.75)	21.54 (6.29)	−1.65	0.05

** *p* < 0.01; * *p* < 0.05.

**Table 2:** Standardized direct and indirect effects.

Link	Direct	Indirect	Total Effect
H1. Maternal Care—adolescents’ Internet addiction problems	−0.08 CI = −0.24 to 0.07	−0.16 ** CI = −0.28 to –0.08	−0.24 ** CI = −0.38 to−0.12
H2. Paternal Care—adolescents’ Internet addiction problems	−0.01 CI = −0.14 to 0.01	−0.09 ** CI = −0.17 to 0.03	−0.11 CI = −0.23 to 0.02
H3: Maternal Care—adolescents’ mental health problems	−0.36 ** CI = −0.49 to −0.23	_	−0.36 ** CI = −0.49 to −0.23
H4: Paternal Care—adolescents’ mental health problems	−0.20 ** CI = −0.33 to −0.08	_	−0.20 ** CI = −0.33 to −0.08
H5: Adolescents’ mental health problems—adolescents’ Internet addiction problems	0.45 ** CI = 0.24 to 0.63	_	0.45 ** CI = 0.24 to 0.63

Note: the confidence intervals (CI) are based on the findings from bootstrapping analysis (1500 samples). ** *p* < 0.01.

## Data Availability

The data presented in this study are available on request from the corresponding author.

## References

[1] Borca G., Bina M., Keller P.S., Gilbert L.R., Begotti T. (2015). Internet use and developmental tasks: Adolescents’ point of view. Comput. Hum. Behav..

[2] Pisano L. (2016). L’identità Virtuale. Teoria E Tecnica Dell’indagine Socio-Psicopedagogica Online.

[3] Lu X., Watanabe J., Liu Q., Uji M., Shono M., Kitamura T. (2011). Internet and mobile phone text-messaging dependency: Factor structure and correlation with dysphoric mood among Japanese adults. Comput. Hum. Behav..

[4] Willoughby T. (2008). A short-term longitudinal study of internet and computer game use by adolescent boys and girls: Prevalence, frequency of use, and psychosocial predictors. Dev. Psychol..

[5] Witt E.A., Massman A.J., Jackson L.A. (2011). Trends in youth’s videogame playing, overall computer use, and communication technology use: The impact of self-esteem and the Big Five personality factors. Comput. Hum. Behav..

[6] Moawad G.E., Ebrahem G.G.S. (2016). The relationship between use of technology and parent-adolescents social relationship. J. Educ. Pract..

[7] Panicker J., Sachdev R. (2014). Relations among loneliness, depression, anxiety, stress and problematic internet use. Int. J. Res. Appl. Nat. Soc. Sci..

[8] Pednekar N., Tung S.S. (2017). Role of parent and peer attachment, and family environment in discriminating between adolescents in low and high problematic internet use groups. Int. J. Indian Psychol..

[9] Andrade A.L.M., Scatena A., de Oliveira Pinheiro B., de Oliveira W.A., Lopes F.M., De Micheli D. (2021). Psychometric Properties of the Smartphone Addiction Inventory (SPAIBR) in Brazilian Adolescents. Int. J. Ment. Health Addict..

[10] Andrade A.L.M., Scatena A., Martins G.D.G., de Oliveira Pinheiro B., da Silva A.B., Enes C.C., Oliveira W.A., Kim D.-J. (2020). Validation of Smartphone Addiction Scale-Short Version (SAS-SV) in Brazilian adolescents. Addict. Behav..

[11] Tang D., Wei F., Qin B., Liu T., Zhou M. (2014). Coooolll: A deep learning system for twitter sentiment classification. Proceedings of the 8th International Workshop on Semantic Evaluation.

[12] Kraut R., Patterson M., Lundmark V., Kiesler S., Mukophadhyay T., Scherlis W. (1998). Internet paradox: A social technology that reduces social involvement and psychological well-being. Am. Psychol..

[13] Spada M.M. (2014). An overview of problematic Internet use. Addict. Behav..

[14] Chou C., Condron L., Belland J.C. (2005). A review of the research on Internet addiction. Educ. Psychol. Rev..

[15] Milani L., Osualdella D., Di Blasio P. (2009). Quality of interpersonal relationships and problematic Internet use in adolescence. CyberPsychol. Behav..

[16] Yao M.Z., Zhong Z.J. (2014). Loneliness, social contacts and internet addiction: A cross-lagged panel study. Comput. Hum. Behav..

[17] Young K.S. (2004). Internet addiction: A new clinical phenomenon and its consequences. Am. Behav. Sci..

[18] Shi X., Wang J., Zou H. (2017). Family functioning and Internet addiction among Chinese adolescents: The mediating roles of self-esteem and loneliness. Comput. Hum. Behav..

[19] Lamb M.E., Lewis C., Bornstein M.H., Lamb M.E. (2005). The Role of Parent-Child Relationships in Child Development. Developmental Science: An Advanced Textbook.

[20] Ong M.Y., Eilander J., Saw S.M., Xie Y., Meaney M.J., Broekman B.F.P. (2018). The influence of perceived parenting styles on socio-emotional development from pre-puberty into puberty. Eur. Child Adolesc. Psychiatry.

[21] Eichenberg C., Schott M., Decker O., Sindelar B. (2017). Attachment Style and Internet Addiction: An Online Survey. J. Med. Internet Res..

[22] Armsden G.C., Greenberg M.T. (1987). The inventory of parent and peer attachment: Individual differences and their relationship to psychological well-being in adolescence. J. Youth Adolesc..

[23] Nickerson A.B., Nagle R.J. (2005). Parent and peer attachment in late childhood and early adolescence. J. Early Adolesc..

[24] Chi X., Lin L., Zhang P. (2016). Internet addiction among college students in China: Prevalence and psychosocial correlates. Cyberpsychol. Behav. Soc. Netw..

[25] Samek D.R., Hicks B.M., Keyes M.A., Bailey J., McGue M., Iacono W.G. (2015). Gene–environment interplay between parent–child relationship problems and externalizing disorders in adolescence and young adulthood. Psychol. Med..

[26] Şenormancı Ö., Şenormancı G., Güçlü O., Konkan R. (2014). Attachment and family functioning in patients with internet addiction. Gen. Hosp. Psychiatry.

[27] Trumello C., Babore A., Candelori C., Morelli M., Bianchi D. (2018). Relationship with Parents, Emotion Regulation, and Callous-Unemotional Traits in Adolescents’ Internet Addiction. BioMed Res. Int..

[28] Cimino S., Cerniglia L., Paciello M., Sinesi S. (2013). A six-year prospective study on children of mothers with eating disorders: The role of paternal psychological profiles. Eur. Eat. Disord. Rev..

[29] Martínez E., Julián A. (2017). Relación entre los estilos educativos parentales o prácticas de crianza y la ansiedad infanto-juvenil: Una revisión bibliográfica. Rev. Española Pedagog..

[30] Babore A., Carlucci L., Cataldi F., Phares V., Trumello C. (2017). Aggressive behaviour in adolescence: Links with self-esteem and parental emotional availability. Soc. Dev..

[31] Martin G., Bergen H.A., Roeger L., Allison S. (2004). Depression in young adolescents: Investigations using 2 and 3 factor versions of the Parental Bonding Instrument. J. Nerv. Ment. Dis..

[32] Yap M.B.H., Pilkington P.D., Ryan S.M., Jorm A.F. (2014). Parental factors associated with depression and anxiety in young people: A systematic review and meta-analysis. J. Affect. Disord..

[33] Heider D., Matschinger H., Bernert S., Alonso J., Brugha T.S., Bruffaerts R., de Girolamo G., Dietrich S., Angermeyer M.C. (2008). Adverse parenting as a risk factor in the occurrence of anxiety disorders: A study in six European countries. Soc. Psychiatry Psychiatr. Epidemiol..

[34] Colonnesi C., Draijer E.M., Stams G.J.J.M., Van der Bruggen C.O., Bögels S.M., Noom M.J. (2011). The relation between insecure attachment and child anxiety: A meta-analytic review. J. Clin. Child Adolesc. Psychol..

[35] Schindler A., Thomasius R., Sack P.M., Gemeinhardt B., Küstner U. (2007). Insecure family bases and adolescent drug abuse: A new approach to family patterns of attachment. Attach. Hum. Dev..

[36] Xu J., Shen L.X., Yan C.H., Hu H., Yang F., Wang L., Kotha S.R., Ouyang F., Zhang L.N., Liao X.P. (2014). Parent-adolescent interaction and risk of adolescent Internet addiction: A population-based study in Shanghai. BMC Psychiatry.

[37] Kormas G., Critselis E., Janikian M., Kafetzis D., Tsitsika A. (2011). Risk factors and psychosocial characteristics of potential problematic and problematic internet use among adolescents: A cross-sectional study. BMC Public Health.

[38] Tokunaga R.S., Rains S.A. (2010). An evaluation of two characterizations of the relationships between problematic internet use, time spent using the internet, and psychosocial problems. Hum. Commun. Res..

[39] Hetzel-Riggin M.D., Pritchard J.R. (2011). Predicting problematic internet use in men and women: The contributions of psychological distress, coping style, and body esteem. Cyberpsychol. Behav. Soc. Netw..

[40] Cao H., Sun Y., Wan Y., Hao J., Tao F. (2011). Problematic internet use in Chinese adolescents and its relation to psychosomatic symptoms and life satisfaction. BMC Public Health.

[41] Raudsepp L., Kais K. (2019). Longitudinal associations between problematic social media use and depressive symptoms in adolescent girls. Prev. Med. Rep..

[42] Wartberg L., Kriston L., Zieglmeier M., Lincoln T., Kammerl R. (2019). A longitudinal study on psychosocial causes and consequences of Internet gaming disorder in adolescence. Psychol. Med..

[43] Richards R., McGee R., Williams S.M., Welch D., Hancox R.J. (2010). Adolescent screen time and attachment to parents and peers. Arch. Pediatrics Adolesc. Med..

[44] Ballarotto G., Volpi B., Marzilli E., Tambelli R. (2018). Adolescent Internet Abuse: A Study on the Role of Attachment to Parents and Peers in a Large Community Sample. Hindawi BioMed Res. Int..

[45] Stavropoulos V., Kuss D., Griffiths M.D., Motti-Stefanidi F. (2016). A longitudinal study of adolescent internet addiction: The role of conscientiousness and classroom hostility. J. Adolesc. Res..

[46] Anderson E.L., Steen E., Stavropoulos V. (2016). Internet use and Problematic Internet Use: A systematic review of longitudinal research trends in adolescence and emergent adulthood. Int. J. Adolesc. Youth.

[47] ISTAT (2013). La Classificazione delle Professioni.

[48] Parker G., Tupling H., Brown L. (1979). A parental bonding instrument. Br. J. Med. Psychol..

[49] Scinto A., Marinangeli M., Kalyvoka A., Daneluzzo E., Rossi A. (1999). The use of the Italian version of the Parental Bonding Instrument (PBI) in a clinical sample and in a student group: An exploratory and confirmatory factor analysis study. Epidemiol. E Psichiatr. Soc..

[50] Goodman R. (1997). The Strengths and Difficulties Questionnaire: A Research Note. J. Child Psychol. Psychiat..

[51] Young K.S. (1998). Internet addiction: The emergence of a new clinical disorder. CyberPsychol. Behav..

[52] Fioravanti G., Casale S. (2015). Evaluation of the psychometric properties of the Italian Internet Addiction Test. Cyberpsychol. Behav. Soc. Netw..

[53] Enders C.K. (2010). Applied Missing Data Analysis.

[54] Anderson J.C., Gerbing D.W. (1988). Structural Equation Modeling in Practice: A Review and Recommended Two-Step Approach. Psychol. Bull..

[55] Westland J. (2010). Erratum: Lower bounds on sample size in structural equation modeling. Electron. Commer. Res. Appl..

[56] Kline R.B. (2011). Principles and Practice of Structural Equation Modeling.

[57] West S.G., Finch J.F., Curran P.J., Hoyle R.H. (1995). Structural Equation Models with Non Normal Variables: Problems and remedies. Structural Equation Modeling: Concepts, Issues, and Applications.

[58] Brown T.A. (2015). Confirmatory Factor Analysis for Applied Research.

[59] Kline R.B. (2016). Principles and Practice of Structural Equation Modeling.

[60] Wong C.-K., Chen Y.-M., Yen C.-F. (2019). Associations of parental bonding and adolescent internet addiction symptoms with depression and anxiety in parents of adolescents with attention deficit/hyperactivity disorder. Arch. Clin. Psychiatry.

[61] Monacis L., de Palo V., Griffiths M.D., Sinatra M. (2017). Exploring Individual Differences in Online Addictions: The Role of Identity and Attachment. Int. J. Ment. Health Addict..

[62] Severino S., Craparo G. (2013). Internet addiction, attachment styles, and social self-efficacy. Glob. J. Psychol. Res..

[63] Cacioppo M., Barni D., Correale C., Mangialavori S., Danioni F., Gori A. (2019). Do Attachment Styles and Family Functioning Predict Adolescents’ Problematic Internet Use? A Relative Weight Analysis. J. Child Fam. Stud..

[64] Musetti A., Terrone G., Schimmenti A. (2018). An exploratory study on problematic internet use predictors: Which role for attachment and dissociation?. Clin. Neuropsychiatry.

[65] Vismara L., Sechi C., Lucarelli L. (2019). Fathers’ and mothers’ depressive symptoms: Internalizing/externalizing problems and dissociative experiences in their adolescent offspring. Curr. Psychol..

[66] Pace U., Schimmenti A., Zappulla C., Di Maggio R. (2013). Psychological variables characterized different type of adolescent gamblers: A discriminant function analysis. Clin. Neuropsychiatry.

[67] Terrone G., Musetti A., Raschielli S., Marino A., Costrini P., Mossi P., Caretti V. (2018). Attachment relationships and internalization and externalization problems in a group of adolescents with pathological gambling disorder. Clin. Neuropsychiatry.

[68] Enns M.W., Cox B.J., Clara I. (2002). Parental bonding and adult psychopathology: Results from the US National Comorbidity Survey. Psychol. Med..

[69] Rosenthal N.L., Kobak R. (2010). Assessing adolescents’ attachment hierarchies: Differences across developmental periods and associations with individual adaptation. J. Res. Adolesc..

[70] Champagne F.A. (2008). Epigenetic mechanisms and the transgenerational effects of maternal care. Front. Neuroendocrinol..

[71] Babore A., De Laurentiis M., Troiano S., Cavallo A., Trumello C., Bramanti S.M. (2020). Depressive symptoms in adolescence. Predictive factors and gender differences [Rischio depressivo in adolescenza Fattori predittivi e differenze di genere]. Psicol. Clin. Dello Svilupp..

[72] Sechi C., Vismara L., Lucarelli L. (2020). Attachment to Parents and Peers and Adolescent Mental Health: The Mediating Role of Alexithymia. Community Ment. Health J..

[73] Gladstone G.L., Parker G.B., Hudson J.L., Rapee R.M. (2005). The role of parenting in the development of psychopathology: An overview of research using the Parental Bonding Instrument. Psychopathology and the Family.

[74] Hayward C., Sanborn K. (2002). Puberty and the emergence of gender differences in psychopathology. J. Adolesc. Health.

[75] Waxler C., Shirtcliff E.A., Marceau K. (2008). Disorders of childhood and adolescence: Gender and psychopathology. Annu. Rev. Clin. Psychol..

[76] Cahill L. (2005). His brain, her brain. Sci. Am..

[77] Leaper C., Bornstein M.H. (2002). Parenting girls and boys. Handbook of Parenting: Children and Parenting.

[78] Cerniglia L., Guicciardi M., Sinatra M., Monacis L., Simonelli A., Cimino S. (2019). The Use of Digital Technologies, Impulsivity and Psychopathological Symptoms in Adolescence. Behav. Sci..

[79] Arpaci I. (2020). Gender differences in the relationship between problematic internet use and nomophobia. Curr. Psychol..

[80] Ha Y.M., Hwang W.J. (2014). Gender differences in internet addiction associated with psychological health indicators among adolescents using a national web-based survey. Int. J. Ment. Health Addict..

[81] Liang L., Zhou D., Yuan C., Shao A., Bian Y. (2016). Gender differences in the relationship between internet addiction and depression: A cross-lagged study in Chinese adolescents. Comput. Hum. Behav..

[82] Lau J.T.F., Walden D.L., Wu A.M.S., Cheng K., Lau M.C.M., Mo P.K.H. (2018). Bidirectional predictions between Internet addiction and probable depression among Chinese adolescents. J. Behav. Addict..

[83] Morita M., Ando S., Kiyono T., Morishima R., Yagi T., Kanata S., Fujikawa S., Yamasaki S., Nishida A., Kasai K. (2021). Bidirectional relationship of problematic Internet use with hyperactivity/inattention and depressive symptoms in adolescents: A population-based cohort study. Eur. Child Adolesc. Psychiatry.

[84] Otsuka Y., Kaneita Y., Itani O., Tokiya M. (2020). Relationship between Internet Addiction and Poor Mental Health among Japanese Adolescents. Iran. J. Public Health.

[85] Wu A.M.S., Li J., Lau J.T.F., Mo P.K.H., Lau M.M.C. (2016). Potential impact of internet addiction and protective psychosocial factors onto depression among Hong Kong Chinese adolescents—Direct, mediation and moderation effects. Compr. Psychiatry.

[86] Wu A.M.S., Lau J.T.F., Cheng K., Law R.W., Tse V.W.S., Lau M.M.C. (2016). Direct and interaction effects of co-existing familial risk factors and protective factors associated with Internet addiction among Chinese students in Hong Kong. J. Early Adolesc..

[87] Babore A., Morelli M., Trumello C. (2021). Italian adolescents’ adjustment before and during the coronavirus disease 2019: A comparison between mothers’ and adolescents’ perception. Br. J. Clin. Psychol..

[88] Trumello C., Lombardi L., Bramanti S.M., Ricciardi P., Candelori C., Cattelino E., Crudele M., Baiocco R., Chirumbolo A., Morelli M. (2021). COVID-19 and home confinement: A study on fathers, father-child relationships, and child adjustment. Child Care Health Dev..

[89] Babore A., Trumello C., Lombardi L., Candelori C., Chirumbolo A., Cattelino E., Baiocco R., Bramanti S.M., Viceconti M.L., Pignataro S. (2021). Mothers’ and Children’s Mental Health During the COVID-19 Pandemic Lockdown: The Mediating Role of Parenting Stress. Child Psychiatry Hum. Dev..

[90] Ballarotto G., Marzilli E., Cerniglia L., Cimino S., Tambelli R. (2021). How does psychological distress due to the COVID-19 pandemic impact on internet addiction and Instagram addiction in emerging adults?. Int. J. Environ. Res. Public Health.

